# Siderophore Cephalosporin Cefiderocol Utilizes Ferric Iron Transporter Systems for Antibacterial Activity against Pseudomonas aeruginosa

**DOI:** 10.1128/AAC.01405-16

**Published:** 2016-11-21

**Authors:** Akinobu Ito, Toru Nishikawa, Shuhei Matsumoto, Hidenori Yoshizawa, Takafumi Sato, Rio Nakamura, Masakatsu Tsuji, Yoshinori Yamano

**Affiliations:** Drug Discovery and Disease Research Laboratory, Shionogi & Co., Ltd., Osaka, Japan

## Abstract

Cefiderocol (S-649266) is a novel parenteral siderophore cephalosporin conjugated with a catechol moiety at the third-position side chain. The *in vitro* activity of cefiderocol against Pseudomonas aeruginosa was enhanced under iron-depleted conditions, whereas that of ceftazidime was not affected. The monitoring of [thiazole-^14^C]cefiderocol revealed the increased intracellular accumulation of cefiderocol in P. aeruginosa cells incubated under iron-depleted conditions compared with those incubated under iron-sufficient conditions. Cefiderocol was shown to have potent chelating activity with ferric iron, and extracellular iron was efficiently transported into P. aeruginosa cells in the presence of cefiderocol as well as siderophores, while enhanced transport of extracellular ferric iron was not observed when one of the hydroxyl groups of the catechol moiety of cefiderocol was replaced with a methoxy group. We conclude that cefiderocol forms a chelating complex with iron, which is actively transported into P. aeruginosa cells via iron transporters, resulting in potent antibacterial activity of cefiderocol against P. aeruginosa.

## INTRODUCTION

Nosocomial infections caused by Gram-negative bacteria are increasingly difficult to treat due to the spread of multidrug-resistant (MDR) strains which are resistant to carbapenems, cephalosporins, aminoglycosides, and quinolones (1–3). In Pseudomonas aeruginosa, resistance to β-lactam antibiotics is due to various resistance factors such as porin OprD deficiency, overproduction of efflux pumps such as MexA-MexB-OprM, overproduction of chromosomal AmpC β-lactamase, and acquisition of exogenous serine or metallo-β-lactamases such as Klebsiella pneumoniae carbapenemase (KPC), New Delhi metallo-β-lactamase (NDM), Verona integron-borne metallo-β-lactamase (VIM), and imipenemase (IMP) (4–13). Since there are very few drugs to treat these highly resistant strains, the development of new antibacterial agents which can be used for the treatment of the infections caused by MDR Gram-negative pathogens is urgently needed.

Iron exists mainly as an insoluble form under physiological conditions, and iron is tightly bound to proteins such as transferrin and lactoferrin in mammalian hosts. The bacteria upregulate the production of small molecules called siderophores, such as pyoverdine of P. aeruginosa, as well as iron uptake systems to scavenge for iron (14–17) and to efficiently incorporate the siderophore-iron complex via iron transporters to obtain iron to survive under iron-depleted conditions, such as in mammalian hosts.

Since the 1980s, numerous attempts to conjugate iron-binding functional groups onto β-lactams have been designed to hijack the iron uptake systems of Gram-negative bacteria and circumvent outer membrane barriers (18–20). However, none of these molecules has been approved for clinical use. Recently, siderophore-conjugated monobactam antibacterial agents, BAL30072, MB-1, MC-1, and SMC-3176 with a hydroxypyridone moiety, have been reported to show potent antibacterial activities against some MDR Gram-negative bacteria, including P. aeruginosa (19, 21, 22), but the development of MB-1 and SMC-3176 has been problematic due to a lack of correlation between *in vitro* activity and *in vivo* efficacy (21–23).

Cefiderocol (S-649266), a novel catechol-substituted siderophore cephalosporin, is structurally different from other recently developed siderophore-conjugated antibacterial agents and has shown potent *in vivo* efficacy, which compares well with *in vitro* activity, in various murine infection models against MDR Gram-negative bacteria, including carbapenem-resistant Enterobacteriaceae, P. aeruginosa, and Acinetobacter baumannii (24–27). We have shown that this potent activity of cefiderocol is partly due to its high stability against various extended-spectrum β-lactamases (ESBLs) and carbapenemases (26, 28). In this study, the underlying active uptake mechanisms of cefiderocol leading to the *in vitro* activity against P. aeruginosa were investigated.

## MATERIALS AND METHODS

### Antibacterial agents.

**FIG 1:**
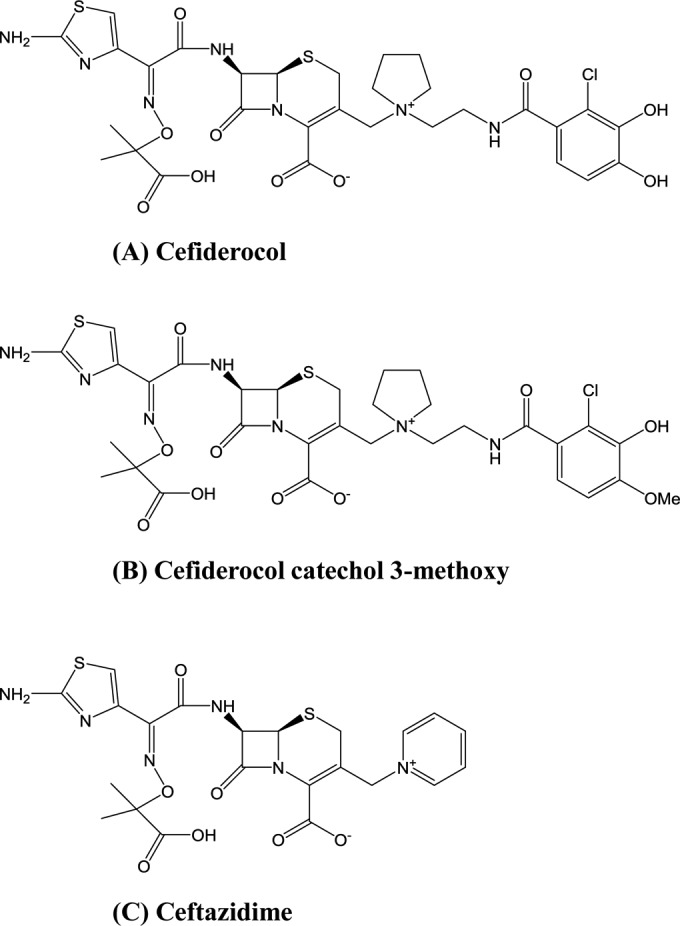
Chemical structures of cefiderocol, cefiderocol catechol 3-methoxy, and ceftazidime.

Cefiderocol (Fig. 1A), cefiderocol catechol 3-methoxy in which one hydroxyl group of the catechol side chain is replaced with a methoxy group (Fig. 1B), and [thiazole-^14^C]cefiderocol were synthesized at the research laboratories of Shionogi & Co., Ltd. (Osaka, Japan). The purity of [thiazole-^14^C]cefiderocol was 95.8%. Ceftazidime was purchased from U.S. Pharmacopeia (Rockville, MD, USA) (Fig. 1C).

### Bacterial strains.

P. aeruginosa PAO1 was kindly provided by Colin Manoil of the University of Washington (29). P. aeruginosa ATCC 27853 was obtained from the American Type Culture Collection (ATCC, VA).

### MIC.

MICs were determined by a broth microdilution method according to the recommendations of the Clinical and Laboratory Standards Institute (CLSI) (30). For the determination of the MIC of cefiderocol, iron-depleted cation-adjusted Mueller-Hinton broth (ID-CAMHB) was used in this study as recommended by the CLSI because MICs of cefiderocol determined in the iron-depleted medium showed a good relationship to *in vivo* efficacy (24–27). ID-CAMHB was prepared as follows. One liter of autoclaved Mueller-Hinton broth (Becton, Dickinson and Company, NJ) was incubated with 100 g of cation binding resin Chelex 100 (Bio-Rad, France) to remove cations, including iron, from the medium and filtered, and the pH was adjusted to 7.3 with hydrochloric acid. The medium was filtered again and supplemented with 20 to 25 mg/liter of Ca^2+^, 10 to 12.5 mg/liter of Mg^2+^, and 0.5 to 1 mg/liter of Zn^2+^, according to CLSI recommendations. The addition of Ca^2+^ and Mg^2+^ was necessary to restore bacterial growth, and the addition of Zn^2+^ was necessary to activate existing metallo β-lactamases which can be important factors involved in multidrug resistance. To evaluate the effect of iron concentration on the MIC, an appropriate amount of iron(III) chloride was added to ID-CAMHB. The iron concentrations in cation-adjusted Mueller-Hinton broth (CAMHB) and ID-CAMHB measured by inductively coupled plasma mass spectrometry (iCAP Qc ICP-MS; ThermoFisher Scientific, MA) were approximately 0.2 mg/liter (3.6 μM) and 0.02 mg/liter (0.4 μM), respectively.

### Uptake of [thiazole-^14^C]cefiderocol into cells.

P. aeruginosa PAO1 was incubated at 37°C for 20 h in iron-depleted medium (ID-CAMHB) and iron-sufficient medium [ID-CAMHB supplemented with 32 μg/ml of ammonium iron(III) citrate, corresponding to approximately 100 μM iron]. The bacteria from each culture were washed and suspended in fresh media to achieve a bacterial suspension at an optical density at 625 nm (OD_625_) of 0.16 to 0.17. After the bacterial suspension in each medium was incubated at 37°C for 10 min, ammonium iron(III) citrate and [thiazole-^14^C]cefiderocol were simultaneously added to the medium at final concentrations of 21.2 μg/ml (approximately 66 μM) and 10 μg/ml (approximately 13 μM), respectively. One-milliliter samples from a total volume of 10 ml were taken at 0.5, 1, 3, 5, and 10 min and filtered through a 0.45-μm-pore-size nitrocellulose membrane filter (Millipore). The filtered samples were washed twice with ice-cold 0.05 M phosphate buffer (pH 7.0), and radioactivity was determined by a liquid scintillation counter (Tri-Carb 3100TR; PerkinElmer, Inc., MA). The amount of [thiazole-^14^C]cefiderocol taken up was calculated by the following equation: uptake amount = (radioactivity × 10^9^)/(SPEC × MW) × (0.1/OD_625_), where radioactivity (expressed as the number of disintegrations per minute [dpm]) was determined by a liquid scintillation counter, SPEC is specific radioactivity (dpm/mg), and MW is molecular weight. Data represent averages of the results obtained by three independent experiments. A *t* test was conducted to determine the significant difference between results at each time point.

### Chelating activity with ferric iron.

Chelating activities of cefiderocol, cefiderocol catechol 3-methoxy, ceftazidime, and pyoverdine, which is the primary siderophore of P. aeruginosa, with ferric iron were determined by the color change of chrome azurol B (CAB) in the presence of cetyltrimethylammonium bromide (CTMA) (31). A solution of 0.5 mM anhydrous piperazine, pH of 5.5, containing 1 mM CAB, 5 mM CTMA, and 4 μM FeCl_3_ was mixed with various concentrations of the test compounds and incubated at room temperature for 60 min. The color changes of the solutions were detected by measuring the *A*_630_ with a spectrophotometer (UV-1200; Shimadzu, Japan). Data represent averages of the results obtained by three independent experiments. A *t* test was conducted to determine the significant difference between results at each concentration.

### Iron uptake into cells monitored by calcein.

Calcein-acetoxymethyl (calcein-AM; Dojindo Molecular Technologies, Inc., Japan), a derivative of calcein, was used as a fluorescent indicator to determine the relative changes of the intracellular iron concentration (32). Once calcein-AM has permeated the cell, the acetoxymethyl group is removed by intracellular esterase, the calcein is trapped inside the cells, and the calcein moiety gives out a green fluorescence. When the iron is incorporated into the cells, the fluorescence of intracellular calcein is quenched by chelating with intracellular iron. P. aeruginosa PAO1 was incubated in ID-CAMHB overnight at 35°C, and calcein-AM was added at a final concentration of 20 μM. At the same time, chloramphenicol was added at a concentration of 97.7 μg/ml to suppress the cell activity. The bacteria would not be killed by this step because the MIC of chloramphenicol against PAO1 is >512 μg/ml. After incubation for 120 min at room temperature under light-shielded conditions, the cells were washed with phosphate buffer, and bacterial density was adjusted to an OD_600_ of 5.0 in Vogel-Bonner minimal medium (33) supplemented with 0.95 mg/ml ammonium iron(III) citrate, corresponding to approximately 3 mM iron. The cells were immediately transferred to a 96-well plate, and green fluorescence intensity was monitored by an EnVision Multilabel Reader (PerkinElmer, Inc., MA) with 492-nm excitation and 535-nm emission. During 300 s of monitoring, 50 μg/ml of the compound was added 90 s after the initiation of the monitoring. Pyoverdine (EMC Microcollections, Germany), one of the intrinsic iron uptake siderophores of P. aeruginosa, was used as a positive control.

### Frequency of resistance.

An overnight culture of P. aeruginosa PAO1 was diluted into fresh medium to yield an inoculum of approximately 1.0 × 10^8^ CFU/ml. Aliquots of 0.1 ml were plated onto Muller-Hinton agar (MHA) (Becton, Dickinson, and Company, NJ) containing 4× or 10× the MICs of the compounds to be tested against the test strain and incubated at 37°C for 48 h. The number of resistant colonies was counted to determine the frequency of resistance.

## RESULTS

### Antibacterial activity of cefiderocol in culture medium with a range of iron concentrations.

MICs of cefiderocol, cefiderocol catechol 3-methoxy, and ceftazidime determined using CAMHB and ID-CAMHB supplemented with a range of ferric iron concentrations are shown in Table 1. The MIC of cefiderocol in ID-CAMHB was 0.031 μg/ml against P. aeruginosa PAO1, while the MIC of cefiderocol catechol 3-methoxy was 8 μg/ml. The treatment by cation binding resin resulted in a 4-fold decrease in the MIC of cefiderocol, and the MIC of cefiderocol was restored by the addition of 0.1 mg/liter of the iron, which was similar to the concentration of the iron in CAMHB. These results suggested that the concentration of the iron is a critical factor for determination of the antibacterial activity of cefiderocol. On the other hand, MICs of cefiderocol catechol 3-methoxy and ceftazidime did not change regardless of the iron concentrations in the medium. These results demonstrated the importance of the catechol moiety for the antibacterial activity of cefiderocol.

**TABLE 1:** MICs of cefiderocol, cefiderocol catechol 3-methoxy, and ceftazidime against P. aeruginosa PAO1 in CAMHB and ID-CAMHB supplemented with a range of ferric iron concentrations

Medium and iron addition (mg/liter)^*a*^	Total iron (mg/liter)	MIC (μg/ml)		
		Cefiderocol	Cefiderocol catechol 3-methoxy	Ceftazidime
CAMHB				
0	0.2	0.125	8	0.5
ID-CAMHB				
0	0.02	0.031	8	0.5
0.01	0.03	0.063	4	0.5
0.03	0.05	0.063	8	0.5
0.05	0.07	0.063	8	1
0.1	0.12	0.125	8	1
0.3	0.32	0.25	8	1
0.5	0.52	0.5	8	1
1	1.02	0.5	8	1
10	10.02	1	8	1

a CAMHB, cation-adjusted Mueller-Hinton broth; ID-CAMHB, iron-depleted CAMHB.

### Uptake of cefiderocol.

**FIG 2:**
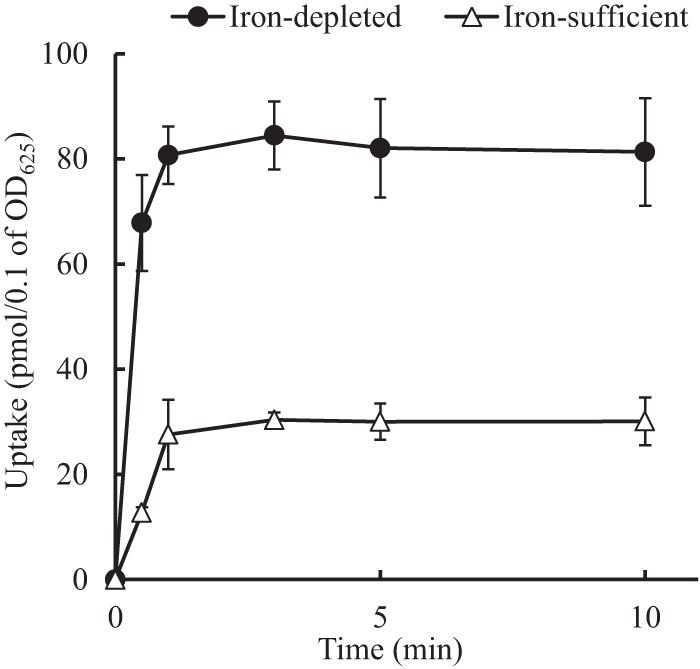
Uptake of [thiazole-^14^C]cefiderocol into P. aeruginosa PAO1. The test strain was incubated under iron-depleted (ID-CAMHB) and iron-sufficient [ID-CAMHB supplemented with 100 μM ammonium iron(III) citrate] conditions overnight. After the culture was washed and incubated at 37°C for 10 min, 66 μM ammonium iron(III) citrate and 10 μg/ml [thiazole-^14^C]cefiderocol were simultaneously added. Samples were taken at 0.5, 1, 3, 5, and 10 min and filtered, and the radioactivity was determined by a liquid scintillation counter. The uptake amounts of [thiazole-^14^C]cefiderocol were significantly different between the cells grown in ID-CAMHB and those grown in CAMHB at all time points (*P* value < 0.05, by *t* test).

The uptake amounts of cefiderocol into P. aeruginosa PAO1 grown in ID-CAMHB and CAMHB supplemented with iron were determined using radiolabeled cefiderocol, [thiazole-^14^C]cefiderocol (Fig. 2). The uptake of [thiazole-^14^C]cefiderocol into the bacterial cells increased immediately after the addition of [thiazole-^14^C]cefiderocol and reached steady state within approximately 1 min. The amount of [thiazole-^14^C]cefiderocol in the cells incubated in ID-CAMHB was significantly higher than that in the cells incubated in CAMHB supplemented with iron at all time points (*P* value < 0.05, by *t* test). These results suggested that the enhanced uptake of cefiderocol into P. aeruginosa cells cultured in ID-CAMHB is associated with the increased activity of cefiderocol in ID-CAMHB.

### Chelating activity of cefiderocol with iron.

**FIG 3:**
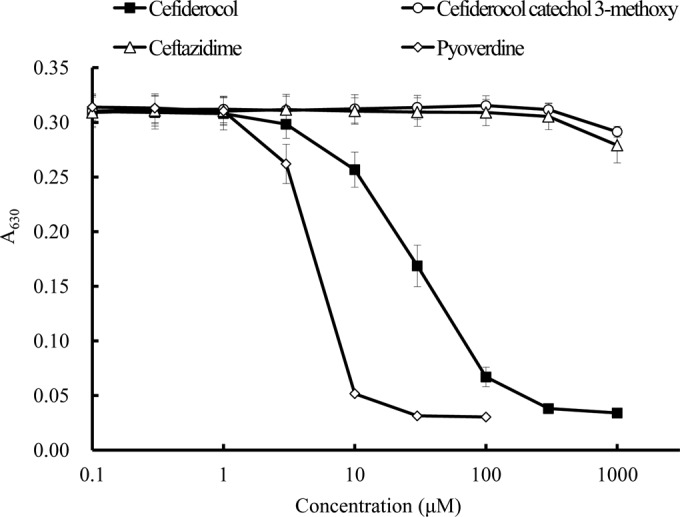
Chelating activity of cefiderocol with ferric iron determined by chrome azurol B. The chelating activity was determined by the color change of chrome azurol B in the presence of cetyltrimethylammonium bromide. The color change was detected by measuring the *A*_630_ with a spectrophotometer. The chelating activities were significantly different between cefiderocol and ceftazidime and between cefiderocol and cefiderocol catechol 3-methoxy at concentrations of 10, 30, 100, 300, and 1,000 μM (*P* value < 0.05, by *t* test). The chelating activities were also significantly different between pyoverdine and ceftazidime and between pyoverdine and cefiderocol catechol 3-methoxy at concentrations of 3, 10, 30, and 100 μM (*P* value < 0.05, by *t* test).

The chelating activity of cefiderocol with ferric iron was determined by using CAB (Fig. 3). Cefiderocol as well as pyoverdine showed chelating activity with ferric iron, while cefiderocol catechol 3-methoxy and ceftazidime, a cephalosporin without a catechol side chain, showed no chelating activity at the range of iron concentrations tested. The chelating activities were significantly different between cefiderocol and ceftazidime and between cefiderocol and cefiderocol catechol 3-methoxy at concentrations of 10, 30, 100, 300, and 1,000 μM (*P* value < 0.05, by *t* test). The chelating activities were also significantly different between pyoverdine and ceftazidime and between pyoverdine and cefiderocol catechol 3-methoxy at the concentrations of 3, 10, 30, and 100 μM (*P* value < 0.05 by *t* test). These results showed that cefiderocol has chelating activity with ferric iron and that the catechol moiety is required for its chelating activity.

### Iron transportation into cells.

**FIG 4:**
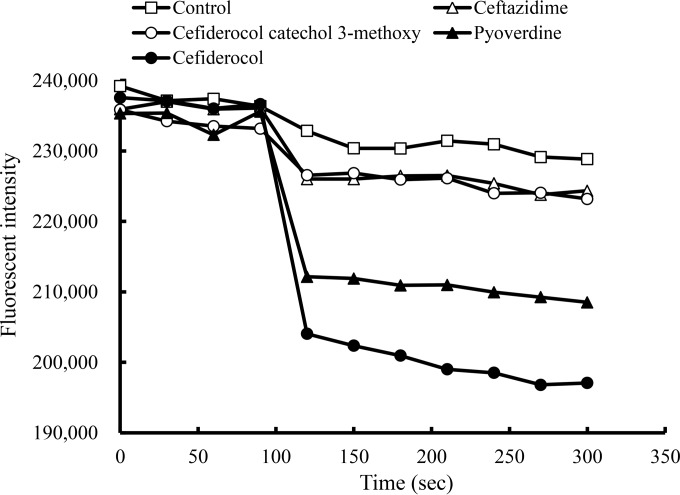
Iron uptake by P. aeruginosa PAO1 detected by calcein fluorescence intensity in the cells. Calcein-AM was added to the overnight culture in iron-depleted medium (ID-CAMHB); the culture was incubated for 120 min, and green fluorescence intensity was monitored by an EnVision Multilabel reader with 492-nm excitation and 535-nm emission. During 300 s of monitoring, 50 μg/ml of the compound was added 90 s after the initiation of the monitoring.

The transport of iron into P. aeruginosa PAO1 cells in the presence of cefiderocol was evaluated by calcein (Fig. 4). Monitoring the fluorescence of intracellular calcein, which is quenched by chelating with intracellular iron, allows us to compare the relative changes in intracellular iron concentrations. The fluorescence intensity of intracellular calcein was quenched immediately after the addition of pyoverdine, indicating that the role of the siderophore to incorporate iron into the cells via iron transport systems could be evaluated by this method. In the same way, fluorescence intensity was also quenched immediately after the addition of cefiderocol, while neither cefiderocol catechol 3-methoxy nor ceftazidime nor abundant extracellular iron itself quenched the intracellular fluorescence intensity. These results indicated that the cefiderocol functions as a siderophore and enters the bacteria via the iron transport systems.

### Frequency of resistance.

The frequencies of resistance of P. aeruginosa PAO1 to cefiderocol were lower than those to ceftazidime, and no resistant colonies appeared when 10× the MIC of cefiderocol was used (Table 2). There were four colonies recovered when 4× the MIC of cefiderocol was used. The MICs of cefiderocol against these colonies were 16 to 32 times the MIC of the parental strain PAO1 although the MICs of ceftazidime against these colonies were not changed compared to the MIC against parental strain PAO1. In the case of the colonies resistant to ceftazidime, MICs of cefiderocol against them did not change. These results showed that cefiderocol did not show cross-resistance with ceftazidime, suggesting that the resistance mechanisms should be different between cefiderocol and ceftazidime. Additional investigation is required to understand the mechanisms of resistance of these colonies against cefiderocol.

**TABLE 2:** Frequency of resistance of P. aeruginosa PAO1 to cefiderocol and ceftazidime

Drug	Frequency of resistance^*a*^ at:	
	4× MIC	10× MIC
Cefiderocol	2.9 × 10^−8^	<7.1 × 10^−9^
Ceftazidime	3.1 × 10^−7^	3.4 × 10^−7^

a Number of appeared colonies/number of inoculum. Here, inoculum was 1.4 × 10^8^, and the frequency of resistance should be 7.1 × 10^−9^ if a resistant colony appears. So, in the case of no resistant colonies, the frequency of resistance should be <7.1 × 10^−9^.

## DISCUSSION

The major findings in this study are as follows: (i) *in vitro* antibacterial activity of cefiderocol is enhanced in iron-depleted medium, and the catechol moiety is important for the antibacterial activity of cefiderocol; (ii) the uptake of cefiderocol by P. aeruginosa is promoted in iron-depleted medium; (iii) the catechol moiety is required for the chelating activity of cefiderocol with iron and is also important for iron transport into P. aeruginosa. These experiments provide direct evidence that cefiderocol utilizes the iron transport system of P. aeruginosa to transport itself effectively by the binding of the catechol moiety with extracellular iron, mimicking the P. aeruginosa siderophore pyoverdine, which results in potent antibacterial activity. The antibacterial activity of cefiderocol as well as the uptake of [thiazole-^14^C]cefiderocol by P. aeruginosa cells was shown to be influenced by the iron concentration in the medium, which is consistent with the fact that the expression of the iron-regulated outer membrane proteins (IROMPs) is upregulated under iron-depleted conditions (34, 35).

The *in vitro* antibacterial activity of cefiderocol is enhanced under iron-depleted conditions, and IROMPs are reported to be upregulated under iron-depleted conditions (17). The result suggested that the uptake of cefiderocol should occur via the IROMPs (17, 36, 37). It was reported that P. aeruginosa infection was not effectively treated in an *in vivo* murine infection model by MB-1, a hydroxypyridone-substituted monobactam, despite low MICs, and that the competition with native siderophores could contribute to the recalcitrance of some P. aeruginosa isolates *in vivo*, which was called adaptation-based resistance (22). Subsequently, cefiderocol has been shown to have potent *in vivo* efficacy against the same strains which showed adaptive resistance to MC-1 (I. Ghazi, M. L. Monogue, M. Tsuji, and D. P. Nicolau, submitted for publication), consistent with earlier *in vivo* experiments using cefiderocol against P. aeruginosa (26, 27). The low frequency of resistance to cefiderocol and the absence of cross-resistance between cefiderocol and the marketed cephalosporins suggest the benefit of cefiderocol. Further detailed studies are required to clarify the differences in mechanisms of action between cefiderocol and other types of siderophore-conjugated β-lactams.

In this study, the underlying mechanisms responsible for the potent *in vitro* activity of cefiderocol against P. aeruginosa were elucidated. Cefiderocol showed potent siderophore activity, and the catechol moiety on its side chain contributed to the chelating activity with ferric iron. Due to its catechol moiety, cefiderocol was transported more efficiently into bacterial cells under free-iron-depleted conditions than under free-iron-sufficient conditions, resulting in enhanced *in vitro* activity against P. aeruginosa.
